# MolFCL: predicting molecular properties through chemistry-guided contrastive and prompt learning

**DOI:** 10.1093/bioinformatics/btaf061

**Published:** 2025-02-08

**Authors:** Xiang Tang, Qichang Zhao, Jianxin Wang, Guihua Duan

**Affiliations:** Hunan Provincial Key Lab on Bioinformatics, School of Computer Science and Engineering, Central South University, Changsha 410083, China; Hunan Provincial Key Lab on Bioinformatics, School of Computer Science and Engineering, Central South University, Changsha 410083, China; Hunan Provincial Key Lab on Bioinformatics, School of Computer Science and Engineering, Central South University, Changsha 410083, China; Hunan Provincial Key Lab on Bioinformatics, School of Computer Science and Engineering, Central South University, Changsha 410083, China

## Abstract

**Motivation:**

Accurately identifying and predicting molecular properties is a crucial task in molecular machine learning, and the key lies in how to extract effective molecular representations. Contrastive learning opens new avenues for representation learning, and a large amount of unlabeled data enables the model to generalize to the huge chemical space. However, existing contrastive learning-based models face two challenges: (i) existing methods destroy the original molecular environment and ignore chemical prior information, and (ii) there is a lack of a prior knowledge to guide the prediction of molecular properties.

**Results:**

In this work, we propose a molecular property prediction framework called MolFCL, which consists of fragment-based contrastive learning and functional group-based prompt learning. Specifically, we introduced fragment–fragment interactions for the first time in the contrastive learning framework and designed a fragment-based augmented molecular graph that integrates the original chemical environment and fragment reactions. Furthermore, we proposed a novel functional group-based prompt learning during fine-tuning, which first incorporates functional group knowledge and the corresponding atomic signals, to improve molecular representation and provide interpretable analyses. The results show that MolFCL outperforms state-of-the-art baseline models on 23 molecular property prediction datasets. Moreover, visualizations show that MolFCL can learn to embed molecules into representations that can distinguish chemical properties. MolFCL can give higher weight to functional groups consistent with chemical knowledge during the prediction of molecular properties, which offers an interpretable ability of the model. Overall, MolFCL is a practically useful tool for molecular property prediction and assists drug scientists in designing drugs more effectively.

**Availability and implementation:**

MolFCL is available at https://github.com/tangxiangcsu/MolFCLSupplementary.

## 1 Introduction

Molecular property prediction is an important component in drug discovery, involving the discovery of promising lead compounds, assessment of clinical safety of drugs, and anticipation of therapeutic efficacy (Khanna 2012, Simões *et al.* 2018). Molecular property measures on wet-lab experiments are reliable, but the process is complex and time-consuming. To speed up drug discovery and reduce the cost, it has become mainstream to construct computer-aided molecular property prediction models over the past few decades (Shen and Nicolaou 2019, Guo *et al.* 2025, David *et al.* 2020).

Molecular representation is a crucial step of molecular property prediction. Traditional molecular representations incorporate expert knowledge, e.g. extended-connectivity fingerprints (ECFP) (Rogers and Hahn 2010), which have become common tools in computational chemistry. With the advancement of deep learning (DL), data-driven molecular representation learning has replaced traditional methods as the mainstream approach. Based on the input form, these end-to-end molecular representations can be divided into sequence-based and graph-based representations. Sequence-based representations (Kusner *et al.* 2017, Gupta *et al.* 2018) take the simplified molecular-input line-entry system (SMILES) strings as input, while graph-based representations employ graph neural networks to learn the molecular topological information which is critical for molecular properties (Dai *et al.* 2016, Kipf and Welling 2016, Gilmer *et al.* 2017, Xu *et al.* 2018, Song *et al.* 2020).

A significant obstacle hindering the development of end-to-end model in molecular property prediction is the scarcity of labeled data (Walters and Barzilay 2021). With the success of molecular generation techniques, a substantial amount of molecular data has been generated in recent years and prompted the application of self-supervised approaches in molecular representation learning. MTL-BERT (Zhang *et al.* 2022) and SmiCLR (Pinheiro *et al.* 2022) leverage SMILES as input to learn molecular representations. In graph learning, N-GRAM (Liu *et al.* 2019) reassembles vertex embeddings in short walks within the graph to construct the graph’s representation. GEM (Fang *et al.* 2022) treats molecules as 3D structures to enhance molecular representations. These methods rely on extensive molecular data to enable models to learn the semantic space of molecules. As a member of self-supervised methods, contrastive learning has already demonstrated profound success in molecular representation learning (You *et al.* 2020, Wang *et al.* 2022a,b), which lies in constructing an augmented molecular graph to maximize the similarity between the augmented molecular graph and the original molecular graph. MolCLR (Wang *et al.* 2022b) utilizes a graph augmentation strategy including atom masking, bond deletion, and subgraph removal to construct augmented molecular graphs for contrastive learning. Considering issues such as activity cliffs, this strategy alter the original chemical environment of the molecule and pose new challenges for molecular representations (Sun *et al.* 2021). Therefore, taking chemical prior knowledge as a guide, it is crucial to construct graph augmentation strategies that do not violate the molecular semantic space.

Another existing issue is the lack of a prior knowledge to guide the prediction of molecular properties. Prompt learning exhibits outstanding performance advantages and generalization by embedding relevant task and domain knowledge (Liu *et al.* 2023). Prompt learning also effectively improve molecular property prediction performance. KANO (Fang *et al.* 2023) employs a knowledge graph of chemical elements and functional groups for prompt learning, while MolCPT (Diao *et al.* 2022) utilizes the cross-attention between molecular motifs and molecular graph vectors for prompt learning. These works separate functional group (motif) feature learning from raw molecular graph representation, neglecting the role of intrinsic atomic signals in functional groups.

Considering these challenges, we propose a molecular property prediction framework, MolFCL, that integrates molecular fragment reactions knowledge into contrastive learning framework by construct augmented graph that do not violating the molecular chemical environment. Besides, MolFCL introduces a novel functional group prompt learning in fine-tuning tasks, in which we further take into account the inherent atomic signals in functional groups to design meaningful prompts for molecular graphs. We conducted a comprehensive evaluation of MolFCL on 23 different molecular property prediction datasets, covering various aspects such as molecular physiology, biophysics, physical chemistry, and ADMET. The experimental results demonstrate the superior performance of our model compared to baselines. Additionally, we explored the representation of molecules and the interpretability of the model, providing multiple perspectives to explain the superior performance of MolFCL.

In summary, the contributions of this article are as follows: (i) we construct a sensible dual-perspective augmented molecular graph for molecules, introducing molecular fragment reactions for the first time in a contrastive learning framework, (ii) we propose a novel functional group prompt learning method that fully leverages functional groups and their intrinsic atomic signals, guiding the molecular property prediction of downstream tasks, and (iii) we conducted extensive experiments to comprehensively evaluate MolFCL and interpret the model from the perspective of functional groups. The experimental results demonstrate the effectiveness and advantages of our approach.

## 2 Materials and methods

### 2.1 Molecular data for pre-training and fine-tuning

In the pre-training phase, we pre-trained MolFCL in advance using 250 000 randomly sampled unlabeled molecules from ZINC15 (Sterling and Irwin 2015). In the fine-tuning phase of molecular property prediction, we used nine datasets from MolecularNet (Wu *et al.* 2018) and 14 smaller datasets from Therapeutics Data Commons (TDC) (Huang *et al.* 2022). These datasets cover molecular data in various domains, including physiology, physical chemistry, biophysics, and ADMET. We applied the Scaffold split to all datasets that divides molecules based on their important scaffolds to provide a better evaluation of the generalization ability of out-of-distribution data samples. For more details of all datasets, please refer to Supplementary Section S1.

### 2.2 Overview of MolFCL

**Figure 1.**
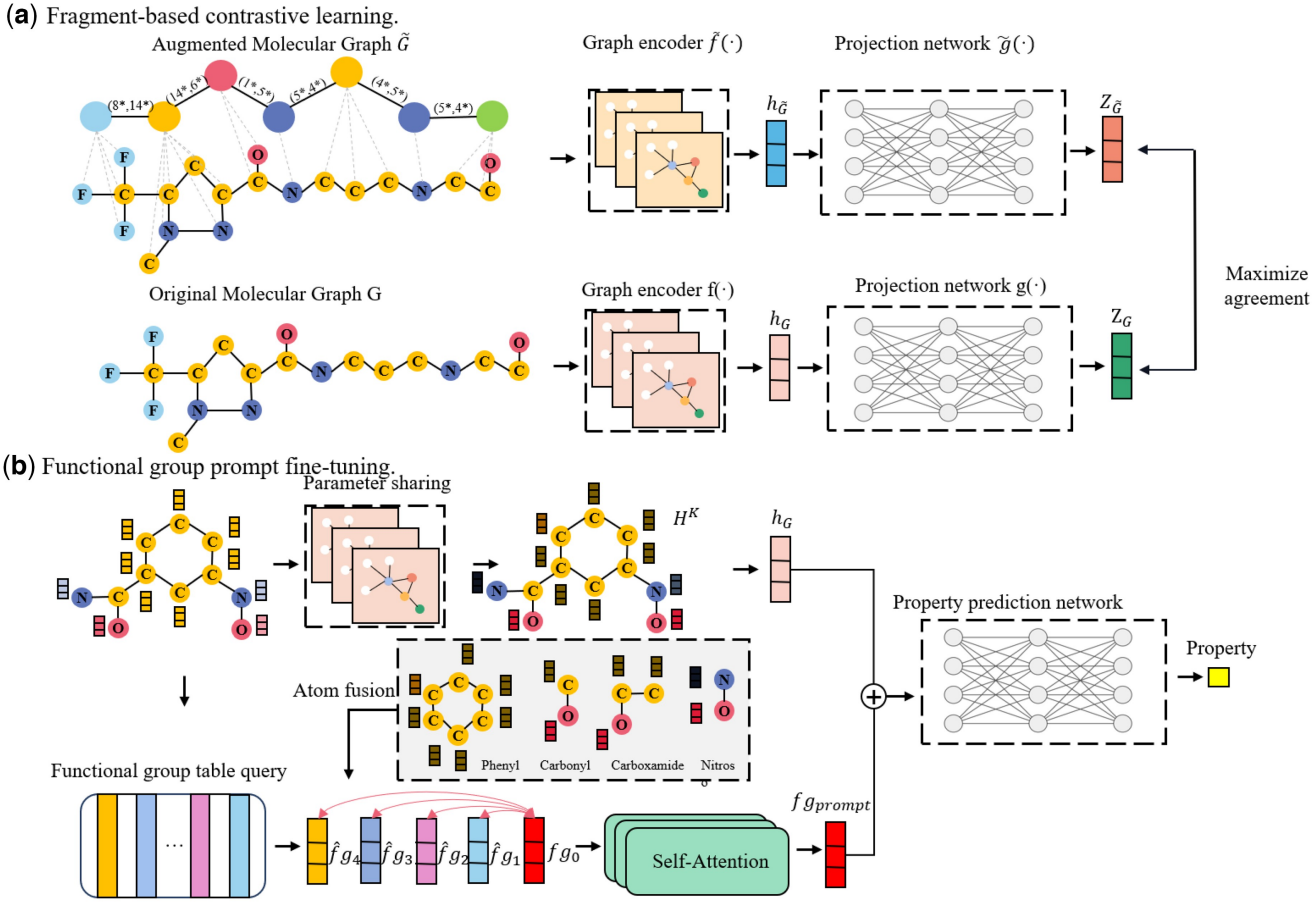
Overview of MolFCL. (a) Fragment-based contrastive learning. Initially, we preserve the original atom–bond relationships in the molecule while employing the BRICS algorithm to break down the molecule. This process establishes connections and reaction relationships between fragments, transforming the original molecular graph *G* into an augmented molecular graph $\tilde{G}$. Subsequently, we pretrain the graph encoder by maximizing consistency among positive pairs and differentiation among negative pairs. (b) Fine-tuning with functional group prompts. We leverage the knowledge of functional groups and their affiliated atomic signals to guide the prediction of model molecular properties.

As shown in Fig. 1, MolFCL consists mainly of two parts: (i) fragment-based contrastive learning, and (ii) functional group prompt fine-tuning. Fragment-based contrastive learning consists of two components: augmented molecular graph and contrastive learning framework, which allows the original molecular graph and the augmented molecular graph to learn from and complement each other. Functional group prompt finetuning aims to leverage knowledge of chemistry to guide molecular property prediction and highlights the role of important functional groups in molecules.

#### 2.2.1 Augmented molecular graph

Prior knowledge in the field of chemistry is crucial for enhancing the representation of molecules. Some researchers have attempted to construct augmented molecular graph by considering chemical similarity between drugs (Wang *et al.* 2022a) or substituting bioisosteres (Sun *et al.* 2021). Given that the substructure plays a crucial role in molecular properties, we attempt to leverage the structures and reactions of molecular fragments to enhance the representation of molecules. As shown in Supplementary Fig. S1, the BRICS algorithm is employed to decompose the molecule into smaller fragments, while preserving the reactions between these fragments. This method aids in understanding reaction processes and structural features within the molecule. Decomposing the molecule into fragments allows for learning the molecular properties at atomic and fragment levels. The atomic-level perspective refers to the traditional molecular graph, where each node represents an atom and each edge represents a chemical bond between atoms. The fragment-level perspective decomposes the molecule into molecular fragments, with nodes and edges representing the interactions between these fragments. By incorporating both perspectives, the augmented molecular graph ensures that both atomic structural information and fragment-level reaction dynamics are preserved. In this context, by preserving the semantic spatial structure of the molecule, the augmented molecular graph design enables the contrastive learning algorithm to focus on capturing intra-molecular reaction knowledge without interference from the spatial arrangement of the molecule. This method enables the model to acquire finer-grained reaction knowledge without losing the original molecular information.

#### 2.2.2 Contrastive learning framework

In contrast learning training, let $G_{i}$ be the original molecular graph and ${\tilde{G}}_{i}$ be the augmented molecular graph. $\left(G_{i},{\tilde{G}}_{i}\right)$ denotes positive pair, while ${\left[\left(G_{i},G_{j}),(G_{i},{\tilde{G}}_{j}),({\tilde{G}}_{i},G_{j}),({\tilde{G}}_{i},{\tilde{G}}_{j}\right)\right]}_{i\neq j}$ denote negative pairs. We capture the representations of molecular graphs by using the graph encoder f(·) for the original graph and $\tilde{f}(\cdot )$ for the augmented molecular graph, formalized as $h_{G_{i}}$ and $h_{{\tilde{G}}_{i}}$. Then, a non-linear projection network g(·) and $\tilde{g}(\cdot )$ are applied to map the representations of the original molecular graph and the augmented molecular graph to a latent space, denoted as $z_{G_{i}}$ and $z_{{\tilde{G}}_{i}}$. Then, the NT-Xent loss function (Pinheiro *et al.* 2022) is employed to maximize the consistency between positive pairs and the dissimilarity between negative pairs, formulated as follows:

(1) $$l_{i}=-\log\frac{e^{\mathrm{sim}(z_{G_{i}},z_{{\tilde{G}}_{i}})/\tau }}{\sum_{k=1}^{2N}1_{\left[k\notin i\right]}e^{\mathrm{sim}(z_{G_{i}},z_{G_{k}})/\tau }},$$

where $\mathrm{sim}(z_{a},z_{b})=\frac{z_{a}^{⊤}z_{b}}{\left||z_{a}||\cdot ||z_{b}|\right|}$ calculates the cosine similarity between vectors and $1_{\left[k\notin i\right]}$ is an indicator function that evaluates to 1 if k≠i, and τ is the temperature parameter.

In the pre-training experiments, MolFCL use the CMPNN (Song *et al.* 2020) model as the original graph encoder f(·) and the method mentioned in PharmHGT (Jiang *et al.* 2023) as the augmented molecular graph encoder $\tilde{f}(\cdot )$, as detailed in Supplementary Section S2. The setup of the projection network g(·) and $\tilde{g}(\cdot )$ are similar to SmiCLR (Pinheiro *et al.* 2022), utilizing a multilayer perceptron (MLP) for the projection.

#### 2.2.3 Functional group prompt finetuning

In finetuning, we share the weights of the original graph encoder f(·) with the downstream property prediction model and finetuning on specific tasks. Specifically, molecular graph G=(V,ε) is fed into the pre-trained encoder f(·) to extract the atomic embedding matrix $H^{K}=\{h_{v}^{K},v\in V\}$ and the graph embedding $h_{G}$.

To introduce functional group knowledge and guide molecular property prediction, we design a novel functional group prompt generator, as shown in Fig. 1b. It generates functional group feature $fg_{\mathrm{prompt}}$ based on the compound’s functional group structure and the input molecular graph. Specifically, we first retrieve the functional groups and their corresponding atoms in the molecule using the open-source chemical analysis toolkit Rdkit, denoted as $S=\{V_{1},\ldots ,V_{m}\}$, where m is the number of functional groups in the molecule, and $V_{i}$ is the set of atoms corresponding to the i-th functional group in the molecule. Then, we query a learnable functional group embedding table $F_{\mathrm{table}}$ to obtain the embedded representations of the relevant functional group $F=\{fg_{1},\ldots ,fg_{m}\}$. After the update of each atomic embedding in the molecular graph, each functional group receives signals from the atoms inside, further enhancing the feature representation of the functional group.

(2) $$f{\hat{g}}_{i}=\sigma \left(W\cdot \mathrm{Concat}(fg_{i},M_{\mathrm{sum}}(V_{i}),M_{\max}(V_{i}))\right),$$

 

(3) $$M_{\mathrm{sum}}(V_{i})=\sum_{v\in V_{i}}h_{v}^{K},$$

 

(4) $$M_{\max}(V_{i})=\mathrm{maxpooling}(h_{v}^{K},v\in V_{i}),$$

where W is trainable parameter.

To emphasize the importance of task-relevant functional groups, we introduce a learnable vector $fg_{0}$ and use a self-attention mechanism (Shang *et al.* 2018) to allow $fg_{0}$ to learn the features of relevant functional groups. Specifically, adding the learnable vector $fg_{0}$ into the functional group set $\hat{F}=\{fg_{0},f{\hat{g}}_{1},\ldots ,f{\hat{g}}_{m}\}$, the self-attention mechanism are as follows:

(5) $$Q=\hat{F}W^{Q},K=\hat{F}W^{k},V=\hat{F}W^{Q},$$

 

(6) $$\tilde{F}=\mathrm{softmax}\left(\frac{QK^{T}}{\sqrt{d}}\right)V,$$

 

(7) $$F_{fg}=\mathrm{LayerNorm}(W\tilde{F}).$$

We obtain the representative prompt vector $fg_{\mathrm{prompt}}=F_{fg}[0,:]$ for the graph G, which learns from functional group of different importance levels. We add it to the vector $h_{G}$ generated by the graph encoder to obtain the final graph embedding vector ${\hat{h}}_{G}$. Finally, we utilize a two-layer MLP to get the prediction scores ${\hat{y}}_{G}$.

(8) $${\hat{h}}_{G}=\mathrm{Concat}(h_{G},\alpha *fg_{\mathrm{prompt}}),$$

 

(9) $${\hat{y}}_{G}=\mathrm{MLP}({\hat{h}}_{G}),$$

where α is a learnable scaling factor.

We defined the objective function for fine-tuning as:

(10) $$\mathrm{mi}n_{\theta^{e},\theta^{p},F_{\mathrm{table}}}\sum_{(G,y_{G})\in T}L({\hat{y}}_{G};y_{G})+\gamma ||F_{\mathrm{table}}|{|}_{2},$$

where $||F_{\mathrm{table}}|{|}_{2}$ is the L2 norm for functional group embedding table $F_{\mathrm{table}}$, γ is the L2 penalty weight. The initialization of molecular encoder $\theta^{e}$ is from the fragment-augmented contrastive learning pre-training. The initialization of the property prediction network $\theta^{p}$ is based on Xavier (Glorot and Bengio 2010) initialization.

### 2.3 Experimental implementation detail

Given the SMILES string of the molecular, we used RDKit to convert it into a molecular graph representation. Following the work of KANO (Fang *et al.* 2023), we initialized the features of atoms and bonds determined by eight attributes, including chirality, atomic mass, atomic degree, etc., and four attributes, including bond type and conjugation. In the augmented molecular graph, we used the BRICS algorithm to extract fragments and corresponding reaction relationships, and used RDKit to extracting fragment and reaction features. Fragment features are primarily determined by seven attributes, including MACCS fingerprints, the number of fragment constituent elements, polar surface area, etc. Reaction features are encoded using one-hot encoding for reaction types. The details are provided in Supplementary Section S3.

In the contrastive learning framework, we set the batch size to 1024 and trained for 50 epochs. We utilize the Adam optimizer with a learning rate of 3e−5 to optimize the NT-Xent loss (Pinheiro *et al.* 2022). The temperature parameter τ is set to 0.1. The projection layer consists of a two-layer MLP with ReLU activation function. During the fine-tuning of downstream tasks, we employ a two-layer MLP network as the property prediction network. For classification tasks, we use Binary Cross Entropy as the loss function for the property prediction. For regression tasks, we utilize Mean Squared Error loss. The training is configured with 100 epochs, a batch size of 256, and we implement an early-stop strategy to prevent overfitting. We perform three independent fine-tuning sessions on the pre-trained model, reporting their average and standard deviation. ROC-AUC is used as the evaluation metric for classification tasks, while Root Mean Square Error (RMSE) serve as the metric for regression tasks. MolFCL is implemented in Pytorch and executed on a one NVIDIA Tesla a100 (40G) GPU. The more details about hyperparameters are provided in Supplementary Section S4.

## 3 Results

### 3.1 MolFCL boosts the performance of property prediction

We compared two types of baselines, the end-to-end models and the pre-trained models. The end-to-end models include GCN (Kipf and Welling 2016), GIN (Xu *et al.* 2018), MPNN (Gilmer *et al.* 2017), DMPNN (Dai *et al.* 2016), and CMPNN (Song *et al.* 2020). The pre-trained models include N-GRAM (Liu *et al.* 2019), Hu et al. (Hu *et al.* 2019), MGSSL (Zhang *et al.* 2021), GEM (Fang *et al.* 2022), GROVER (Rong *et al.* 2020), Molformer-XL (Ross *et al.* 2022), and KANO (Fang *et al.* 2023). To ensure a fair comparison, we followed the experimental setup of previous works, which involved conducting experiments using three independent runs on MolecularNet datasets and five independent runs on TDC datasets. For details of all baselines are displayed in Supplementary Section S7.

To comprehensively evaluate the performance of MolFCL, we tested its capabilities on 14 datasets from MolecularNet related to physiology, physical chemistry, biophysics, and quantum mechanics. Tables 1 and 2 displays the results of MolFCL and baselines. In classification task, our model consistently outperforms baselines on 7 out of 8 datasets, achieving the second-best performance on the Sider dataset. Compared to the best end-to-end model CMPNN (Song *et al.* 2020), MolFCL achieved a significant improvement, specifically gaining at least 6.57% improvement across all 8 datasets. This indicates that using the same molecular encoder, our proposed molecular contrastive learning framework provides a more robust parameter initialization for the encoder. In comparison with the best pre-trained method KANO (Fang *et al.* 2023), our approach surpasses it on all eight datasets, particularly with an improvement of 2.1% on the BBBP dataset, highlighting the effectiveness of the fragment-enhanced graph strategy. In regression tasks related to physical chemistry and quantum mechanics, MolFCL significantly outperforms other methods on four out of six datasets, achieving the second-best performance on the Lipophilicity and QM9 dataset. In contrast to CMPNN, MolFCL shows an average improvement of 23.4%. Remarkably, MolFCL outperforms KANO by 4.8%, 8.5%, 4.6%, and 2.4% on the ESOL (Delaney 2004), FreeSolv (Mobley and Guthrie 2014), QM7, and QM8 datasets, respectively.

**Table 1.** Performance comparison of MolFCL with baselines on classification datasets.^a^

	ROC-AUC, higher is better↑							
Dataset	BBBP	Tox21	Toxcast	Sider	Clintox	Bace	MUV	HIV
Moleculars	2039	7831	8575	1427	1478	1513	93 807	41 127
GCN^b^	0.718 ± 0.009	0.709 ± 0.003	0.650 ± 0.061	0.536 ± 0.003	0.625 ± 0.028	0.716 ± 0.020	0.716 ± 0.040	0.740 ± 0.030
GIN^b^	0.658 ± 0.045	0.740 ± 0.008	0.667 ± 0.015	0.573 ± 0.016	0.580 ± 0.044	0.701 ± 0.054	0.718 ± 0.025	0.753 ± 0.019
MPNN^b^	0.913 ± 0.041	0.808 ± 0.024	0.691 ± 0.030	0.595 ± 0.030	0.879 ± 0.054	0.815 ± 0.010	0.757 ± 0.013	0.770 ± 0.014
DMPNN^b^	0.919 ± 0.030	0.759 ± 0.007	0.637 ± 0.002	0.570 ± 0.007	0.906 ± 0.006	0.852 ± 0.006	0.786 ± 0.014	0.771 ± 0.005
CMPNN^b^	0.927 ± 0.017	0.801 ± 0.016	0.708 ± 0.013	0.616 ± 0.003	0.898 ± 0.008	0.867 ± 0.002	0.790 ± 0.020	0.782 ± 0.022
N-GRAM^b^	0.912 ± 0.003	0.769 ± 0.027	–	0.632 ± 0.005	0.875 ± 0.027	0.791 ± 0.013	0.769 ± 0.007	0.787 ± 0.004
Hu et.al^b^	0.708 ± 0.015	0.787 ± 0.004	0.657 ± 0.006	0.627 ± 0.008	0.726 ± 0.015	0.845 ± 0.007	0.813 ± 0.021	0.799 ± 0.007
MGSSL^b^	0.705 ± 0.011	0.764 ± 0.004	0.641 ± 0.007	0.618 ± 0.008	0.807 ± 0.021	0.797 ± 0.008	0.787 ± 0.015	0.795 ± 0.011
GEM^b^	0.888 ± 0.004	0.781 ± 0.004	0.686 ± 0.002	0.632 ± 0.015	90.3 ± 0.007	87.9 ± 0.011	0.753 ± 0.015	0.813 ± 0.021
GROVER^b^	0.868 ± 0.022	0.803 ± 0.020	0.568 ± 0.034	0.612 ± 0.025	0.703 ± 0.137	0.824 ± 0.036	0.673 ± 0.018	0.682 ± 0.011
MolCLR^b^	0.733 ± 0.010	0.741 ± 0.053	0.659 ± 0.021	0.612 ± 0.036	0.898 ± 0.027	0.828 ± 0.007	0.789 ± 0.023	0.774 ± 0.006
MolCLR (CMPNN)^b^	0.724 ± 0.007	0.784 ± 0.026	0.691 ± 0.012	0.597 ± 0.034	0.880 ± 0.040	0.850 ± 0.024	0.745 ± 0.021	0.778 ± 0.055
Molformer-XL	0.901 ± 0.027	0.838 ± 0.010	0.716 ± 0.011	**0.676 ± 0.006**	0.928 ± 0.022	0.929 ± 0.011	0.735 ± 0.008	0.833 ± 0.011
KANO^b^	0.960 ± 0.016	0.837 ± 0.013	0.732 ± 0.016	0.652 ± 0.008	0.944 ± 0.003	0.931 ± 0.021	0.837 ± 0.023	0.851 ± 0.022
MolFCL	**0.981 ± 0.002**	**0.841 ± 0.015**	**0.741 ± 0.015**	0.660 ± 0.017	**0.955 ± 0.015**	**0.933 ± 0.010**	**0.843 ± 0.019**	**0.855 ± 0.020**

a The best result is shown in bold and the second best result is underlined.

b The results are derived from KANO (Fang *et al.* 2023).

**Table 2.** Performance comparison of MolFCL with baselines on regression datasets.^a^

	RMSE, lower is better↓			MAE, lower is better↓		
Dataset	ESOL	Freesolv	Lipophilicity	QM7	QM8	QM9
Moleculars	1128	642	7160	4300	21 786	133 885
GCN^b^	1.431 ± 0.050	2.870 ± 0.135	0.712 ± 0.049	122.9 ± 2.2	0.0366 ± 0.000	0.00835 ± 0.00001
GIN^b^	1.452 ± 0.020	2.765 ± 0.180	0.850 ± 0.071	124.8 ± 0.7	0.0371 ± 0.001	0.00824 ± 0.00004
MPNN^b^	1.167 ± 0.430	1.621 ± 0.952	0.672 ± 0.051	111.4 ± 0.9	0.0148 ± 0.001	0.00522 ± 0.00003
DMPNN^b^	1.050 ± 0.008	1.673 ± 0.082	0.683 ± 0.016	103.5 ± 8.6	0.0156 ± 0.001	0.00514 ± 0.00001
CMPNN^b^	0.798 ± 0.112	1.570 ± 0.442	0.614 ± 0.029	75.1 ± 3.1	0.0153 ± 0.002	0.00405 ± 0.00002
N-GRAM^b^	1.100 ± 0.030	2.510 ± 0.191	0.880 ± 0.12	125.6 ± 1.5	0.0320 ± 0.003	0.00964 ± 0.00031
Hu et.al^b^	1.100 ± 0.006	2.764 ± 0.002	0.739 ± 0.003	113.2 ± 0.6	0.0215 ± 0.001	0.00922 ± 0.00004
GEM^b^	0.813 ± 0.028	1.748 ± 0.114	0.674 ± 0.022	60.0 ± 2.7	0.0163 ± 0.001	0.00562 ± 0.00007
GROVER^b^	1.423 ± 0.288	2.947 ± 0.615	0.823 ± 0.010	91.3 ± 1.9	0.0182 ± 0.001	0.00719 ± 0.00208
MolCLR^b^	1.113 ± 0.023	2.301 ± 0.247	0.789 ± 0.009	90.9 ± 1.7	0.0185 ± 0.013	0.00480 ± 0.00003
MolCLR(CMPNN)^b^	0.911 ± 0.082	2.021 ± 0.133	0.875 ± 0.003	89.8 ± 6.3	0.0179 ± 0.001	0.00475 ± 0.00001
Molformer-XL	0.698 ± 0.066	1.486 ± 0.231	0.621 ± 0.047	71.8 ± 1.8	0.0165 ± 0.000	0.00571 ± 0.00002
KANO^b^	0.670 ± 0.019	1.142 ± 0.258	**0.566 ± 0.007**	56.4 ± 2.8	0.0123 ± 0.000	**0.00320 ± 0.00001**
MolFCL	**0.638 ± 0.024**	**1.045 ± 0.160**	0.601 ± 0.034	**53.8 ± 5.9**	**0.0120 ± 0.000**	0.00374 ± 0.00014

a The best result is shown in bold and the second best result is underlined.

b The results are derived from KANO (Fang *et al.* 2023).

As shown in Tables 1 and 2, MolFCL performed superiorly on 13 of the 14 datasets compared to the large chemical language model MolFormer-XL. It is noteworthy that MolFormer-XL was pre-trained on 1.1 billion unlabeled molecules from PubChem and ZINC datasets, while MolFCL only utilized 250 000 unlabeled molecules. This indicates that introducing fragment reactions during the pre-training phase improves the model’s ability to learn from the data and provide more robust representations. MolCLR (CMPNN) is variant model that the parameters of CMPNN are generated by the pre-training used in MolCLR. Compared to MolCLR (CMPNN), our method achieves an average superior improvement of 12.8%. This indicates that under the same encoder structure, our pre-training approach provides the model with a better initialization. Intuitively, guiding the model with chemical knowledge leads to better learning of molecular representations.

To comprehensively compare the generalization of MolFCL and the best baselines CMPNN and KANO on datasets with more complex properties and smaller scales, we selected nine datasets from the TDC database with a data size smaller than 1000 for experimentation. As shown in Supplementary Table S6, MolFCL achieved the best performance in seven out of nine datasets, and second-best performance in the Caco2 (Wang *et al.* 2016a) and hERG (Wang *et al.* 2016b) datasets. This indicates that on datasets with smaller scales and more complex attributes, MolFCL can provide more useful information, greatly assisting in addressing tasks with limited labels.

In summary, MolFCL outperforms other state-of-the-art models in several tasks, validating the effectiveness of pre-training with fragment reactions and functional group prompt fine-tuning.

### 3.2 Visualization on molecular representations

Molecules with the same scaffold often exhibit similar molecular representations and properties, while those with different scaffolds show distinct representations and properties. We visualized molecular representations with different scaffolds using t-SNE. We selected seven common scaffolds from BBBP and Tox21, where molecules with the same scaffold share the same color. As depicted in Supplementary Fig. S3, no pre-training models (i.e. randomly initialized model parameters) perform poorly in distinguishing molecules with different scaffolds. In contrast, MolFCL effectively discriminates molecules with various scaffolds and exhibits lower DB indexes. These visualizations further confirm the sensitivity of MolFCL to molecular scaffolds guided by fragment reactions and functional groups.

**Figure 2.**
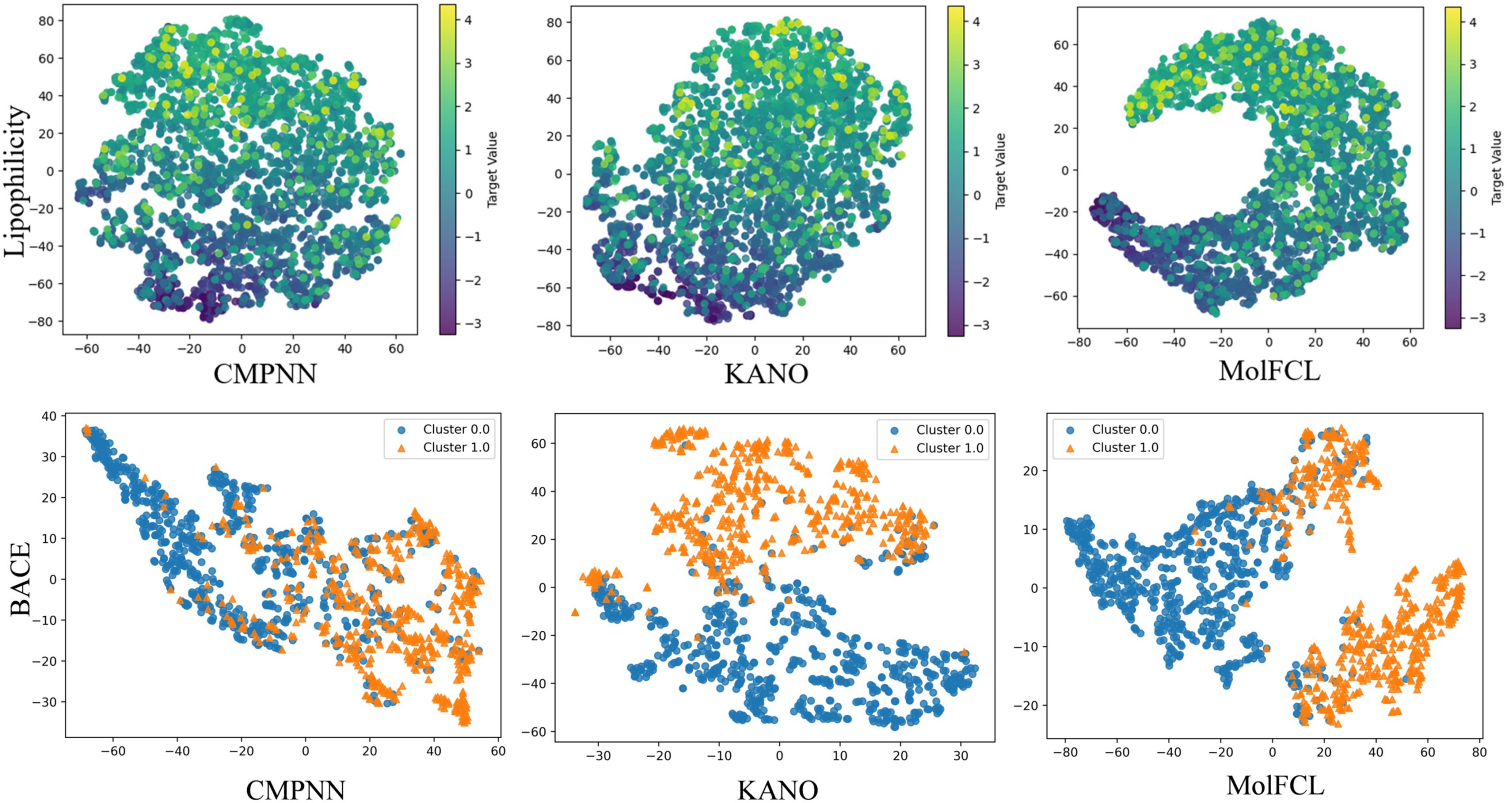
Visualization of molecular features. We employ t-SNE to visualize the features of compounds in Lipophilicity and BACE datasets.

To investigate whether MolFCL can extract effective molecular representations, we employed t-SNE (Van Der Maaten 2014) for visualization of the representations extracted by MolFCL with the best baselines CMPNN and KANO, as shown in Fig. 2. Note that in the t-SNE visualization, we use the final graph embedding representation from Equation (8) to compute t-SNE, which means that this layer’s features integrate molecular embeddings and functional group embeddings. We visualized molecular representations for the Lipophilicity and BACE (Subramanian *et al.* 2016) datasets. For the Lipophilicity dataset measures the ability of drugs to dissolve in a lipid environment. Compared to other methods, MolFCL can learn smoother molecular representations within molecules with continuous property values. For the BACE dataset, which provides a set of binary labeling results for human β-secretase 1 inhibitors, triangles indicate molecules with inhibitory effects, and solid dots indicate non-inhibitory molecules. As depicted in Fig. 2, MolFCL consistently outperforms in distinguishing molecules through molecular representations and exhibits lower Davies–Bouldin (DB) indexes. This suggests that MolFCL can learn more effective representations of molecules. For visualizations of more datasets, please refer to Supplementary Figs S4 and S5 in Supplementary Section S9.

We note that MolFCL on the BACE dataset, as shown in Supplementary Fig. S6A, our model divides the yellow points (positive samples) into two clusters, where we define the lower cluster as Cluster 1 and the upper cluster as Cluster 2. We apply the BRICS algorithm to fragment Cluster 1 and Cluster 2, respectively. By counting the fragment occupancy, we find that aromatic rings have a higher occupancy in Cluster 1 as shown in Supplementary Fig. S6B. The work of Galeana-Ascencio *et al.* (2023) states that in BACE prediction, the aromatic ring is more likely to interact with the hydrophobic pocket around the Ile109 residue in BACE-1, thus acting as an inhibitor. As a result, Cluster 1 has a more pronounced difference in the distribution of features compared to Cluster 2 with respect to the negative samples.

### 3.3 Statistical interpretability of the model

Functional group prompt learning primarily guides molecular property prediction by providing crucial substructures of functional groups. In our study, we pre-defined the structures of 82 functional groups. Analyzing the weights assigned by MolFCL to different functional groups helps us understand how the model predicts specific molecules.

**Figure 3.**
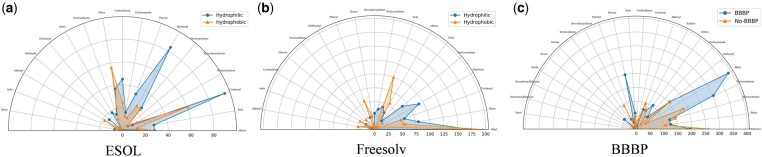
Statistical investigation of interpretability for the functional group prompt. (a) Analysis on the ESOL dataset. (b) Analysis on the Freesolv dataset. (c) Analysis on the BBBP dataset.

The statistical analysis on the ESOL dataset is presented in Fig. 3a. Solid dots indicate hydrophilic molecules, while triangles indicate hydrophobic molecules. Only functional groups with MolFCL assigned weights >0.15 are included in the figure. For hydrophilic molecules, MolFCL tends to focus on Hydroxyl, Carbonyl, and Carboalkoxy group. In contrast, for hydrophobic molecules, MolFCL places greater emphasis on Ether and other common hydrophobic groups. These captured functional groups are all consistent with the expected chemical outcomes.

The statistical analysis on the FreeSolv dataset is presented in Fig. 3b. Solid dots represent molecules with lower hydration free energy. Triangles represent molecules with higher hydration free energy. Only functional groups with weights >0.1 are included in the figure. For low hydration free energy molecules, MolFCL tends to focus on Hydroxyl groups, which chemically enhance molecular hydrophilicity, thereby reducing molecular hydration free energy. In contrast, for high hydration free energy molecules, MolFCL places greater emphasis on hydrophobic groups such as Halogens, including Chloro atoms, and Phenyl groups, which elevate the compound’s hydration free energy.

The statistical analysis on the BBBP dataset is presented in Fig. 3c. Solid dots indicate that molecules can penetrate the blood-brain barrier, while triangles indicate that molecules cannot penetrate the blood–brain barrier. Only functional groups with weights >0.1 are included in the figure. For molecules that are permeable, MolFCL focus on Tertiary Amine, Alkyl, and Phenyl groups. Tertiary Amine enhances lipid solubility, while Alkyl and Phenyl groups are typically hydrophobic, aiding in the penetration of drugs through the blood-brain barrier. Conversely, for non-penetrative molecules, MolFCL places greater emphasis on Hydroxyl groups. Hydroxyl groups increase the compound’s hydrophilicity, and since the blood–brain barrier is primarily composed of lipids, it exhibits strong selectivity for molecules with higher hydrophilicity, making them less likely to penetrate the blood–brain barrier.

Exploring interpretability demonstrates the significance of functional group structures in determining molecular properties. Careful study of each functional group information helps drug scientists screen compounds, identify pivotal structures within compounds, and enhance drug design practices.

### 3.4 Case study

We also selected four molecules from the ESOL and Tox21 datasets for detailed property prediction analysis. As shown in Supplementary Fig. S7, we visualize the weights assigned by MolFCL to different functional groups of interest. (i) On the ESOL dataset, for hydrophilic molecules, MolFCL allocates higher weights to Amino and Hydroxyl groups, which are common hydrophilic groups. Conversely, for hydrophobic molecules, MolFCL assigns higher weights to Phenyl and Ather groups, which are common hydrophobic groups. (ii) On the Tox21 dataset, for non-toxic molecules, MolFCL assigns higher weights to Carboxyl and Hydroxyl groups, which align with chemical knowledge as detoxifying groups. In contrast, for toxic molecules, MolFCL allocates higher weights to Bromine, Chlorine and Aromatic Amine groups, among others, which align with chemical knowledge as toxic groups. Through case study, it becomes more intuitive to observe the chemically reasonable explanations provided by MolFCL, facilitating a transparent exploration of the sources of attribution signals in molecular properties.

### 3.5 Ablation study

To demonstrate the value of the individual modules in MolFCL, we conducted an ablation study to assess their effectiveness. Specifically, we set up w/o MolFCL pretrain, w/o MolFCL atom message, w/o MolFCL FP and the full model for comparison:

- *w/o MolFCL pretraining*: We mask the fragment-based contrastive learning process of the MolFCL and preserve the model structure including functional group prompt learning.
- *w/o MolFCL atom message*: In this setting, the atomic signal in the functional group prompt learning will not be available, and the rest of the settings remain unchanged.
- *w/o MolFCL FP*: In this setting, we block the entire functional group prompt learning, but retain the pre-training process.

Supplementary Table S7 shows the ablation experiments performed on the nine downstream benchmark datasets. The results indicate that the complete model consistently performs better, demonstrating that both fragment-based contrastive learning and functional group prompt learning can enhance the accuracy of molecular property prediction, validating the effectiveness of each component. Specifically, compared to the original CMPNN, the ablated components always perform better, further verifying the effectiveness of different component combinations. It is worth mentioning that the absence of fragment-based contrastive learning process significantly decreases the performance of the model. This suggests that the reasonable introduction of structure and reaction knowledge provides a better initialization parameter for the downstream training of the model and mitigates the overfitting problem caused by the missing training data to some extent.

Moreover, the training times of three independent runs are detailed in Supplementary Table S8. There was no significant difference in test run times for the variant models, with 100 molecules taking ∼1 s on the NVIDIA Tesla a100 (40G) GPU.

We similarly performed ablation experiments for prompt learning. We used ECFP chemical descriptors to replace functional group vectors for prompt learning, the details and experiments of which are in Supplementary Section S13. As shown in Supplementary Table S9, functional group-based prompt learning achieves a very good performance improvement over ECFP prompt. This improvement arises from the ability of functional groups to provide finer-grained information combinations. Furthermore, our carefully designed model architecture enables the selective emphasis on key functional group information while attenuating the influence of less critical groups. In contrast, ECFP descriptors encode information from the entire molecule at a coarser granularity, making it difficult to bridge the gap between pretraining tasks and downstream fine-tuning tasks.

## 4 Conclusion

In this work, we propose a prior knowledge-guided molecular property prediction model MolFCL, which first combine the fragment-based contrastive learning and functional group-based prompt learning. Specifically, we introduced molecular fragment reactions in self-supervised contrastive learning method. We utilize the BRICS algorithm to decompose compounds, retain reaction information between them, and construct augmented molecular graphs that respect chemical space. MolFCL learns chemical representations of molecules through contrastive learning between positive and negative pairs. Furthermore, we propose a novel functional group prompt learning to address the limitation of dropping node features in existing prompt learning methods, and guide molecular property prediction in downstream tasks. This approach captures substructures relevant to downstream tasks, offering new avenues for interpretability in drug design and optimization facilitated by MolFCL. The evaluation shows MolFCL achieved outstanding performance across 23 benchmark datasets.

While MolFCL exhibits notable performance advantages, it still has some limitations. For instance, the effective construction of augmented molecular graphs relies on predefined BRICS rules. When a compound’s structure falls outside these rules, the molecule cannot be segmented, and MolFCL is unable to form a coherent augmented molecular graph. In our future work, we will collect more knowledge about functional groups and molecular reactions to construct a complete molecular splitting scheme. In addition, we employ a randomly initialized learnable table for functional group generation during prompt learning. Unifying the set of functional groups in the pre-training and fine-tuning phases and migrating the pre-training features of the functional groups to the downstream tasks will further narrow the gap between the pre-training task and the downstream tasks and improve the utilization of the pre-training features in the downstream task, and integrate the chemical knowledge.

## Supplementary Material

btaf061_Supplementary_Data
