# Supplementary material for: MolFCL: predicting molecular properties through chemistry-guided contrastive and prompt learning
Source: Bioinformatics. 2025 Feb 8;41(2):btaf061. doi: 10.1093/bioinformatics/btaf061 (PMC11878793; doi:10.1093/bioinformatics/btaf061)
Supplement: btaf061_Supplementary_Data [file btaf061_supplementary_data.docx]

**MolFCL: predicting molecular properties through chemistry-guided contrastive and prompt learning (Supplementary Data)**

XiangTang^1,†^, Qichang Zhao^1,†^, Jianxin Wang^1^ and Guihua Duan^1,*^

^1^Hunan Provincial Key Lab on Bioinformatics, School of Computer Science and Engineering, Central South University, Changsha,410083, China. ^†^The authors wish it to be known that, in their opinion, the first two authors should be regarded as Joint First Authors.

* Contact: [duangh@csu.edu.cn](mailto:duangh@csu.edu.cn)

1. **Details of molecular datasets**

Supplementary Table S1 describes the benchmark datasets used in our work. It includes information on the task types, evaluation metrics, and the number of molecules. These datasets are sourced from MolecularNet and cover a range of molecular physiology, biophysics, physical chemistry, and quantum mechanics.

**Supplementary Table S1.** The description of the benchmark datasets used in our work.

| Task type | metric | Category | Dataset | Tasks | Molecules |
| --- | --- | --- | --- | --- | --- |
| Classification | ROC-AUC | Physiology | BBBP | 1 | 2039 |
|  |  |  | Tox21 | 12 | 7831 |
|  |  |  | Toxcast | 617 | 8575 |
|  |  |  | SIDER | 27 | 1427 |
|  |  |  | Clintox | 2 | 1478 |
|  |  | Biophysics | BACE | 1 | 1513 |
|  |  |  | MUV | 17 | 93807 |
|  |  |  | HIV | 1 | 41127 |
| Regression | RMSE | Physical | ESOL | 1 | 1128 |
|  |  |  | Freesolv | 1 | 642 |
|  |  |  | Lipophilicity | 1 | 4200 |
|  | MAE | Quantum mechanics | QM7 | 1 | 7160 |
|  |  |  | QM8 | 12 | 21786 |
|  |  |  | QM9 | 3 | 133885 |

**Classification datasets**

- BBBP contains binary labels for the blood-brain barrier permeability of over 2000 compounds.
- Tox21 provides qualitative toxicity measurements and stress response pathways for 7831 compounds against 12 different targets, including nuclear receptors.
- ToxCast provides toxicology data for a large compound library based on in vitro high-throughput screening. This includes qualitative results from over 600 experiments for 8576 compounds.
- Sider provides measurements for adverse effects of 1427 marketed and approved drugs, categorizing drug side effects into 27 organ system classes.
- Clintox provides data for 1478 drug compounds with known chemical structures, encompassing two classification tasks: (1) clinical trial toxicity and (2) FDA approval status.
- Bace provides quantitative ($IC_{50}$) and qualitative (binary label) binding results for 1513 compounds as inhibitors of human $\beta$-secretase 1 (BACE-1).
- The Maximum Unbiased Validation (MUV) dataset is a benchmark dataset for PubChem BioAssay containing 17 tasks for approximately 90,000 compounds designed to validate virtual screening techniques.
- The HIV dataset comes from the Drug Therapy Program (DTP) HIV antiviral screen and contains more than 40,000 compounds that inhibit HIV replication. It categorizes compounds into confirmed inactive (CI), confirmed active (CA), and confirmed moderately active (CM).

**Regression datasets**

- ESOL is a small dataset containing water solubility data for 1128 compounds.
- Freesolv provides experimental and computed hydration-free energy for small molecules in water.
- Lipophilicity provides experimental results for the octanol/water partition coefficient (log D at pH 7.4) for 4200 compounds.
- The QM7 dataset, a subset of the GDB-13 database, contains nearly 100 million stable and synthesizable organic molecules containing up to seven “heavy” atoms (C, N, O, S). The 3D coordinates of the most stable conformations of these molecules and electronic properties such as atomization energies, HOMO/LUMO eigenvalues, etc., have been determined using ab initio density functional theory (PBE0/tier2 basis set).The QM7 dataset is used to predict these electronic properties.
- The QM8 dataset from MoleculeNet comprises over 21,000 molecules with up to eight heavy atoms, offering four quantum mechanical properties calculated using advanced methods like TDDFT and CC2, focusing on electronic spectra and excited state energies.
- The QM9 dataset is a comprehensive collection providing geometric, energetic, electronic, and thermodynamic properties for 134 thousand stable organic molecules with up to nine heavy atoms, modeled using density functional theory.

To better evaluate the advantages and limitations of MolFCL and the best baselines, CMPNN and KANO on datasets with smaller scales and more complex molecular properties, we selected all datasets from Therapeutics Data Commons with a data size smaller than 1000. Supplementary Table S2 provides a brief description of these datasets, including task types, evaluation metrics, and the number of molecules.

**Supplementary Table S2.** The description of the smaller and more complex benchmark datasets used in our work.

| Task Type | Metric | Category | Dataset | Tasks | Molecules |
| --- | --- | --- | --- | --- | --- |
| Classification | ROC-AUC | ADMET | Bioavailability | 1 | 640 |
|  |  |  | HIA | 1 | 578 |
|  |  |  | CYP2C9_Substrate | 1 | 666 |
|  |  |  | CYP2D6_Substrate | 1 | 664 |
|  |  |  | CYP3A4_Substrate | 1 | 667 |
|  |  |  | hERG | 1 | 648 |
|  |  |  | DILI | 1 | 475 |
| Regression | RMSE |  | Caco2 | 1 | 906 |
|  |  |  | Half_Life | 1 | 667 |

**Classification datasets**

- Bioavailability: Oral bioavailability is defined as “the rate and extent to which the active ingredient or active moiety is absorbed from a drug product and becomes available at the site of action”.
- HIA: When a drug is orally administered, it needs to be absorbed from the human gastrointestinal system into the bloodstream of the human body. This ability of absorption is called human intestinal absorption (HIA) and it is crucial for a drug to be delivered to the target.
- CYP2C9_Substrate: CYP P450 2C9 plays a major role in the oxidation of both xenobiotic and endogenous compounds. Substrates are drugs that are metabolized by the enzyme.
- CYP2D6_Substrate: CYP2D6 is primarily expressed in the liver. It is also highly expressed in areas of the central nervous system, including the substantia nigra.
- CYP3A4_Substrate: CYP3A4 is an important enzyme in the body, mainly found in the liver and the intestine. It oxidizes small foreign organic molecules (xenobiotics), such as toxins or drugs, so that they can be removed from the body.
- hERG: Human ether-à-go-go related gene (hERG) is crucial for the coordination of the heart's beating. Thus, if a drug blocks the hERG, it could lead to severe adverse effects. Therefore, reliable prediction of hERG liability in the early stages of drug design is quite important to reduce the risk of cardiotoxicity-related attritions in the later development stages.
- DILI: Drug-induced liver injury (DILI) is a fatal liver disease caused by drugs and it has been the single most frequent cause of safety-related drug marketing withdrawals for the past 50 years (e.g. iproniazid, ticrynafen, benoxaprofen). This dataset is aggregated from the U.S. FDA’s National Center for Toxicological Research.

**Regression datasets**

- Caco2: The human colon epithelial cancer cell line, Caco-2, is used as an in vitro model to simulate the human intestinal tissue. The experimental result on the rate of drug passing through the Caco-2 cells can approximate the rate at which the drug permeates through the human intestinal tissue.
- Half_Life: The half-life of a drug is the duration for the concentration of the drug in the body to be reduced by half. It measures the duration of the actions of a drug. This dataset is from and we obtain the deposited version under CHEMBL assay 1614674.

Across all benchmark datasets, we employed the Scaffold split, which is a more challenging partitioning method. Scaffold split involves splitting molecules with different scaffolds into different subsets, ensuring that the molecules in the test set have structural differences from the training set.

1. **Molecular graph encoder**
2. **Original Graph Encoder**

MolFCL uses the CMPNN model as the original graph encoder to encode molecular graphs into feature vectors. CMPNN improves the embedded representation of the graph by enhancing the message interaction between nodes and their corresponding edges. Let $G=\left( V,\varepsilon\right)$ be the molecular graph, $h_{v}^{0}$ be the node features, and $h_{e_{u,v}}^{0}$ be the edge features. First, each node $v \in V$ receives representations from incoming edges to obtain a message vector.

$$m_{v}^{k}=\sum_{u\in N\left( v \right)} h_{e_{u,v}}^{k-1}maxpooling\odot\left( h_{e_{u,v}}^{k-1},u\in N\left( v \right) \right),$$

where $m_{v}^{k}$ represents the message vector of node $v$, $e_{u,v}$ represents the edge from source node $u$ to target node $v$. $h_{e_{u,v}}^{k-1}$ is the edge hidden state transmitted to node and $\odot$ represents the element-wise multiplication.

Then, the current hidden state $h_{v}^{k-1}$ of the node and the message vector $m_{v}^{k}$ are aggregated, updating the current node's hidden state.

$$h_{v}^{k}=\sigma\left( W^{k}\cdot Concat\left( h_{v}^{k-1},m_{v}^{k} \right) \right),$$

where $W^{k}$ represents the MLP operation, and $\sigma$ denotes the rectified linear unit (ReLU) activation.

Next, we obtain the updated message vector $m_{e_{u,v}}^{k}$ for edges as follows,

$$m_{e_{u,v}}^{k}=h_{u}^{k}-h_{e_{v,u}}^{k-1}.$$

The hidden feature vector of edges $h_{e_{u,v}}^{k}$ are as follows,

$$h_{e_{u,v}}^{k}=\sigma\left( x_{e_{u,v}}+W\cdot m_{e_{u,v}}^{k} \right).$$

After $K$ iterations, a final round of interaction is conducted to obtain the node messages $m_{v}$.

$$m_{v}=\sum_{u\in N\left( v \right)} h_{e_{u,v}}^{K}\odot maxpooling\left( h_{e_{u,v}}^{K},u\in N\left( v \right) \right).$$

Then, $m_{v}$ along with the final node representation $h_{v}^{K}$ and the initial node features $h_{v}^{0}$, are concatenated to obtain the final node representation $h_{v}$. Finally, a readout operator is applied to obtain the representation of the entire molecular graph $h_{G}$.

$$h_{G}=\frac{1}{\left| V \right|}\sum_{v\in V} GRU\left( Concat\left( m_{v},h_{v}^{K},h_{v}^{0} \right) \right),$$

where GRU stands for Gated Recurrent Unit.

1. **Augmented molecular graph encoder**

In the augmented molecular graph, we view the molecule as a two-perspective structure. The first is the atomic-level perspective, with atoms as the nodes, and the second is the fragment-level perspective, with molecular fragments as the nodes. We independently run message passing and aggregation in each perspective, updating node and edge features. Then, we aggregate features of two perspectives using a multi-head attention mechanism to obtain the molecular representation of the augmented molecular graph. Note that the following formulas simultaneously update on two perspectives.

Each node $v\in V$ initially receives a message vector $m_{v}^{k}$ from incoming edges, and then concatenates these vectors.

$$m_{v}^{k}=Concat\left( h_{e_{u,v}}^{k-1},\forall u\in N\left( v \right) \right).$$

The current hidden state $h_{v}^{k-1}$ of each node,$v$ is aggregated with the message vector $m_{v}^{k}$ using a multi-head attention mechanism, allowing nodes to focus on the features of edges.

$$h_{v}^{k}=multihead\left( h_{v}^{k-1},m_{v}^{k},m_{v}^{k} \right)+h_{v}^{k-1},$$

$$multihead\left( Q,K,V \right)=Concat\left( head_{1},\ldots,head_{H} \right)W^{O},$$

$$head_{i}=Attention\left( QW_{i}^{Q},KW_{i}^{K},VW_{i}^{V} \right),$$

$$Attention\left( Q,K,V \right)=softmax\left( \frac{QK^{T}}{\sqrt{d_{k}}} \right)V,$$

where H represents the number of heads, $W_{i}^{Q}\in R^{d_{m}\times d_{k}},W_{i}^{K}\in R^{d_{m}\times d_{k}},W_{i}^{V}\in R^{d_{m}\times d_{k}},d_{k}=d_{m}/H$.

Next, we obtain the updated message vector $m_{e_{u,v}}^{k}$ for edges.

$$m_{e_{u,v}}^{k}=h_{v}^{k}-h_{e_{u,v}}^{k-1}.$$

Finally, we update the hidden feature vector for edges.

$$h_{e_{u,v}}^{k}=\sigma\left( x_{e_{u,v}}+W\cdot m_{e_{u,v}}^{k} \right).$$

After $K$ iterations, in the final interaction round, we obtain the last node message $m_{v}$.

$$m_{v}=\sum_{u\in N\left( v \right)} h_{e_{u,v}}^{K}.$$

Then, we concatenate it with the final node representation $h_{v}^{K}$ and the initial node feature $h_{v}^{0}$, resulting in the ultimate node representation $h_{v}$.

$$h_{v}=Concat\left( m_{v},h_{v}^{K},h_{v}^{0} \right).$$

Running independently for each perspective, we obtain atomic perspective features $h_{v}^{atom}$ and a molecular fragment perspective features $h_{v}^{frag}$. By utilizing a multi-head attention mechanism, we aggregate atomic features and fragment features, to get the overall hidden feature $h_{\tilde{v}}$ of the augmented molecular graph. Finally, employing a readout operator, we obtain the representation of the augmented molecular graph $h_{\tilde{G}}$ .

$$h_{\tilde{v}}=multihead\left( h_{v}^{atom},h_{v}^{frag},h_{v}^{frag} \right),$$

$$h_{G}=\frac{1}{\left| V \right|}\sum_{v\in V} GRU\left( h_{\tilde{v}} \right).$$

1. **Initial features in original and augmented molecular graphs**

We utilize RDKit to initialize node and edge features for both the original and augmented graphs. Supplementary Table S3 provides specific chemical considerations taken into account during this process.

**Supplementary Table S3.** Atom and bond features in the original graph.

| Feature type | Features | Description | Size |
| --- | --- | --- | --- |
| Atom | Atom type | Types of atoms (e.g., C, N, O, F) | 110 |
|  | Degree | Number of bonds involving the atom | 6 |
|  | Atom mass | Atomic mass fraction | 1 |
|  | Is Aromatic | Is the atom in the aromatic group? | 1 |
|  | Hybridization | Sp,Sp2,Sp3,Sp3d,Sp3d2 | 5 |
|  | TotalNumHs | Number of hydrogen atoms bonded to the atom | 5 |
|  | ChiralTag | Unspecified, Tetrahedral CW/CCW, or other | 5 |
|  | Formal Charge | Integer electronic charge assigned to an atom | 5 |
| Bond | Bond type | Single, double, triple, aromatic bonds | 4 |
|  | Conjugated | Conjugation of bonds | 1 |
|  | Is Ring | Whether the bond is part of a ring | 1 |
|  | Stereo | None, any, E/Z or cis/trans | 6 |

The augmented graph involves a dual perspective. The first layer, initialized at the atomic level, is detailed in Supplementary Table S3, while the fragment-level feature initialization is outlined in Supplementary Table S4.

**Supplementary Table S4.** Fragment and react features in the augmented graph.

| Feature type | Features | Description | Size |
| --- | --- | --- | --- |
| Fragment | TPSA | Fragment polar surface area | 1 |
|  | MolLogP | LogP Value | 1 |
|  | Heavy Atom MolWt | The average molecular weight of hydrogen-ignored fragments | 1 |
|  | IsRing | Whether the fragment contains a ring | 1 |
|  | RingNum | Number of rings in the fragment | 1 |
|  | ElementNum | Types and numbers of atoms in the fragment | 10 |
|  | MaccsKeys | MACCS fingerprints | 167 |
| React | React feature | Whether there is a reaction between fragments and the type of reaction | 34 |

1. **Experimental implementation detail**

Given the SMILES string of the molecular, we used RDKit to convert it into a molecular graph representation. Following the work of KANO, we initialized the features of atoms and bonds determined by eight attributes, including chirality, atomic mass, atomic degree, etc, and four attributes, including bond type and conjugation. In the augmented molecular graph, we used the BRICS algorithm to extract fragments and corresponding reaction relationships and used RDKit to extract fragment and reaction features. Fragment features are primarily determined by seven attributes, including MACCS fingerprints, the number of fragment constituent elements, polar surface area, etc. Reaction features are encoded using one-hot encoding for reaction types. The details are provided in Supplementary Tables S3 and S4.

In the contrastive learning framework, we set the batch size to 1024 and trained for 50 epochs. We utilize the Adam optimizer with a learning rate of 3e-5 to optimize the NT-Xent loss. The temperature parameter $\tau$ is set to 0.1. The projection layer consists of a two-layer MLP with a ReLU activation function.

During the fine-tuning of downstream tasks, to ensure a fair comparison, we followed the experimental setup of previous works, which involved conducting experiments using three different splits of train/val/test data. For classification tasks, we use Binary Cross Entropy as the loss function for the property prediction. For regression tasks, we utilize Mean Squared Error loss. The training is configured with 100 epochs, a batch size of 256, and we implement an early-stop strategy to prevent overfitting. We perform three independent fine-tuning sessions on the pre-trained model, reporting their average and standard deviation. ROC-AUC is used as the evaluation metric for classification tasks, while RMSE serves as the metric for regression tasks. MolFCL is implemented in Pytorch and executed on one NVIDIA Tesla a100 (40G) GPU. More details about hyperparameters are provided in Supplementary Table S5.

**Supplementary Table S5:** The hyper-parameter setting of MolFCL in pre-training and finetuning process

| Hyper-parameter | Pre-training | Finetuning on  MolecularNet | Finetuning on TDC |
| --- | --- | --- | --- |
| Hidden size | 300 | 300 | 300 |
| Dropout rate | 0.1 | [0,0.1] | 0.1 |
| Batch size | 1024 | 256 | 64 |
| Number of attention heads | 6 | [2,4,7] | 4 |
| Predictor layer | NA | 2 | 2 |
| Predictor hidden size | NA | 300 | 300 |
| Epochs | 50 | [100,300] | [100] |
| Lr | 3e-5 | [1e-4,1e-3] | [3e-5,3e-4] |
| Lr decay | ExponentialLR | NoamLR | NoamLR |
| Weight decay | 0 | 0 | 0 |
| Early stop patience | NA | [10,15,30, NA] | 15 |

1. **Augmented Graph Construction**


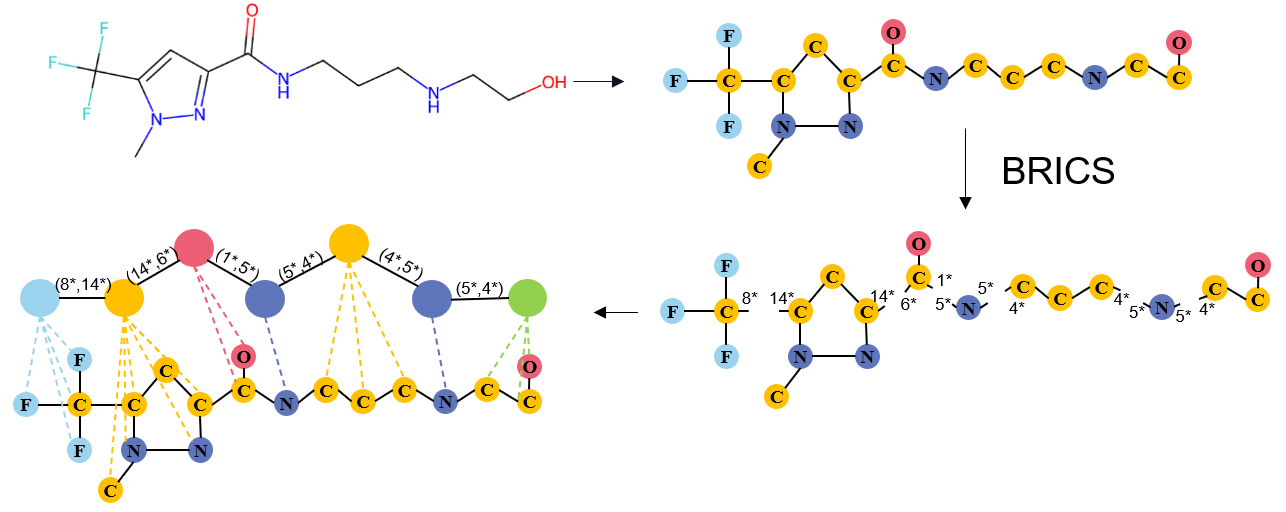


**Supplementary Figure S1.** Augmented graph construction, where {(8*,14*), (14*,6*), (1*,5*), (5*,4*), (4*,5*), (5*,4*)} represents different rule-based fragment marker points that can form new bonds by linking adjacent atoms. BRICS defines a total of 16 fragment structure rules.

1. **Hyper-Parameter Analysis**

In our proposed approach, there are three key hyperparameters, the fine-tuning learning rate, the number of self-attention layers of functional group prompt learning and L2 penalty weight γ. We study the effects of learning rate in the range of {1e-2,5e-3,1e-3,5e-4,1e-4}, self-attention layers in the range of {2,3,4,5,6,7} and L2 penalty weight γ in the range of {0.1, 1, 10, 50, 100}. Generally, the learning rate has the most direct impact on performance, so we determined its optimal value first. After fixing the learning rate, we tuned the number of self-attention layers and the L2 penalty weight (γ) to improve the model’s robustness. Specifically, we prioritized fixing the number of self-attention layers before adjusting γ. Supplementary Fig.S2 shows the effects of parameters on the MolFCL on three different scale regression task datasets. We can see that when learning rate takes 1e-3, self-attention layers take 7 and L2 penalty weight γ takes 0.1, MolFCL achieves the best result.


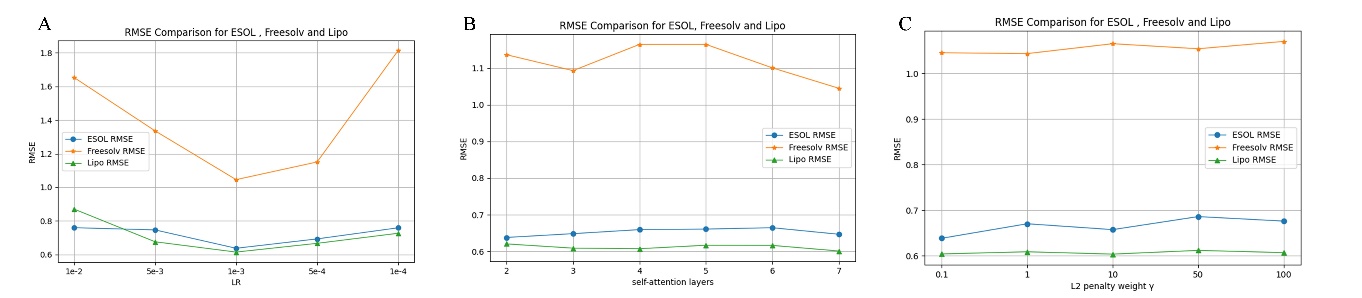


**Supplementary Figure S2.** The effects of hyperparameter setting on MolFCL in regression task datasets. (A) Functional group prompt fine-tuning learning rate. (B) The number of self-attention layers. (C) L2 penalty weight γ.

1. **The detail of all baselines.**

To demonstrate the effectiveness of MolFCL in molecular property prediction, we compare it with supervised and self-supervised baseline models, evaluating MolFCL's performance from various perspectives.

**Supervised learning baselines**

- GCN and GIN are two common graph neural network models.
- MPNN, DMPNN, and CMPNN are models commonly used for molecular property prediction tasks, known for their improvements in the message-passing mechanism.

**Self-supervised learning baselines**

- Hu et al. and GROVER incorporate both node-level and graph-level knowledge in the pretex task.
- N-GRAM constructs the representation of a graph for molecular property prediction by assembling vertices through the shortest paths in the graph.
- MGSSL is a method that employs motif-level prediction tasks to learn the structure of molecular graphs.
- GEM incorporates molecular geometry information and utilizes molecular geometry augmentation to learn molecular representations.
- MolCLR employs a generic graph augmentation algorithm for contrastive learning to learn molecular representations.
- Molformer-XL is a model based on Transformer, pre-trained on a large unlabeled molecular corpus to learn universal molecular representations.
- KANO is a contrastive learning method that incorporates a knowledge graph of chemical elements. It introduces functional group prompt learning during the fine-tuning phase.

1. **Additional study on more complex properties datasets**

**Supplementary Table S6.** Performance comparison of MolFCL with baselines on datasets from Therapeutics Data Commons.

| Models | Classification (ROC-AUC, higher is better↑) | | | | | | | Regression (MAE, lower is better↓) | |
| --- | --- | --- | --- | --- | --- | --- | --- | --- | --- |
|  | Biao | HIA | $CYP2C9_{sub}$ | $CYP2D6_{sub}$ | $\mathrm{CYP}3A4_{sub}$ | hERG | DILI | Caco2 | HalfLife |
| CMPNN | 0.619（0.013） | 0.881（0.006） | 0.615（0.049） | 0.758（0.010） | 0.559（0.009） | 0.784（0.039） | 0.901（0.013） | 0.449（0.001） | 24.809（2.921） |
| KANO | 0.652（0.028） | 0.975（0.008） | 0.595（0.078） | 0.778（0.023） | 0.598（0.015） | **0.844（0.006）** | 0.897（0.010） | **0.387（0.035）** | 24.193（2.256） |
| MolFCL | **0.661（0.019）** | **0.978（0.006）** | **0.627（0.005）** | **0.781（0.039）** | **0.618（0.039）** | 0.823（0.005） | **0.912（0.007）** | 0.425（0.013） | **23.779（1.949）** |

1. **Additional visualization on molecular representation**

Molecules with the same scaffold often exhibit similar molecular representations and properties, while those with different scaffolds show distinct representations and properties. We visualized molecular representations with different scaffolds using t-SNE. We selected seven common scaffolds from BBBP and Tox21, where molecules with the same scaffold share the same color. As depicted in Supplementary Fig. S3, No pre-training models (i.e., randomly initialized model parameters) perform poorly in distinguishing molecules with different scaffolds. In contrast, MolFCL effectively discriminates molecules with various scaffolds and exhibits lower DB indexes. These visualizations further confirm the sensitivity of MolFCL to molecular scaffolds guided by fragment reactions and functional groups.


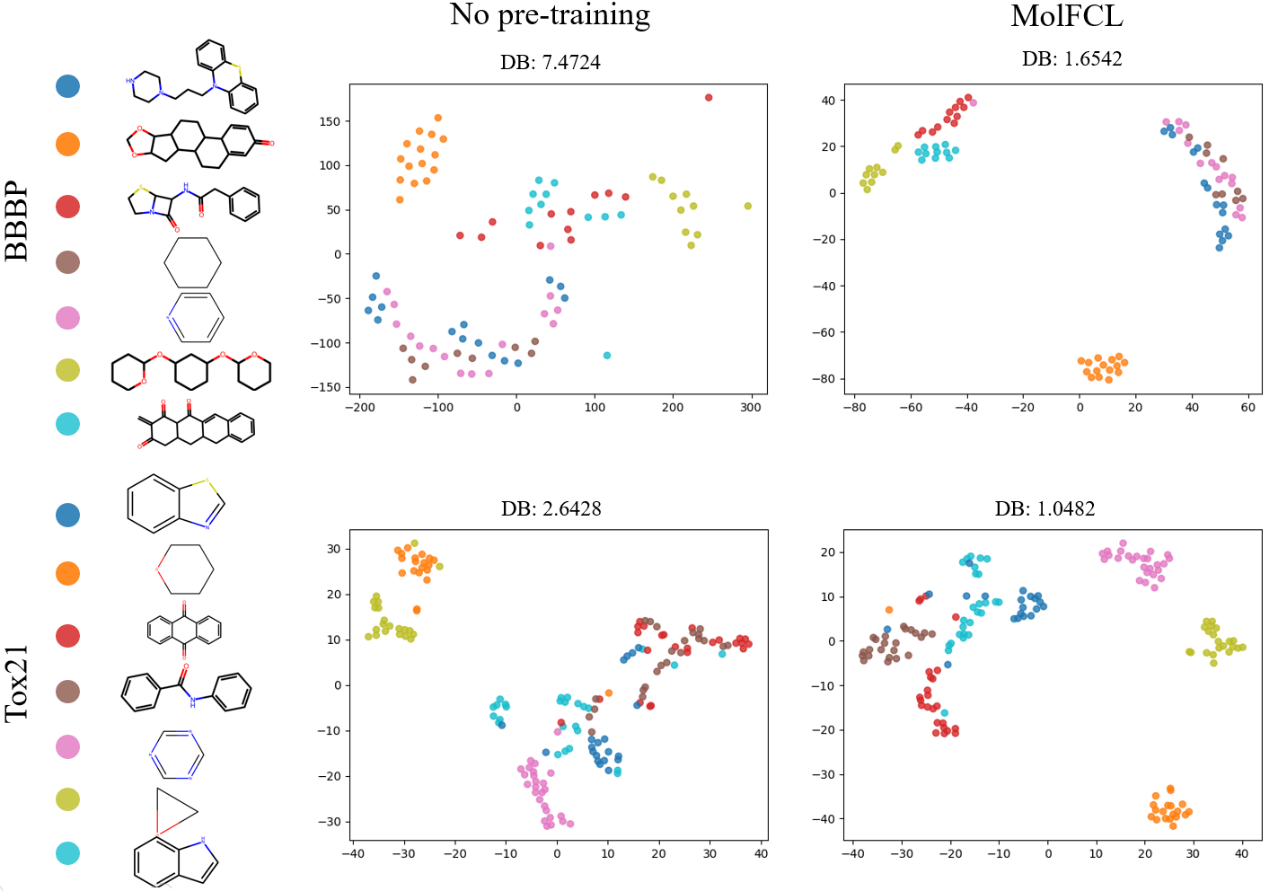


**Supplementary Figure S3.** We utilize t-SNE to showcase molecular representations under different molecular scaffolds, where similar representations correspond to compounds with the same scaffold. Different colors indicate distinct scaffolds, with a smaller Davies-Bouldin (DB) index suggesting better clustering separation.


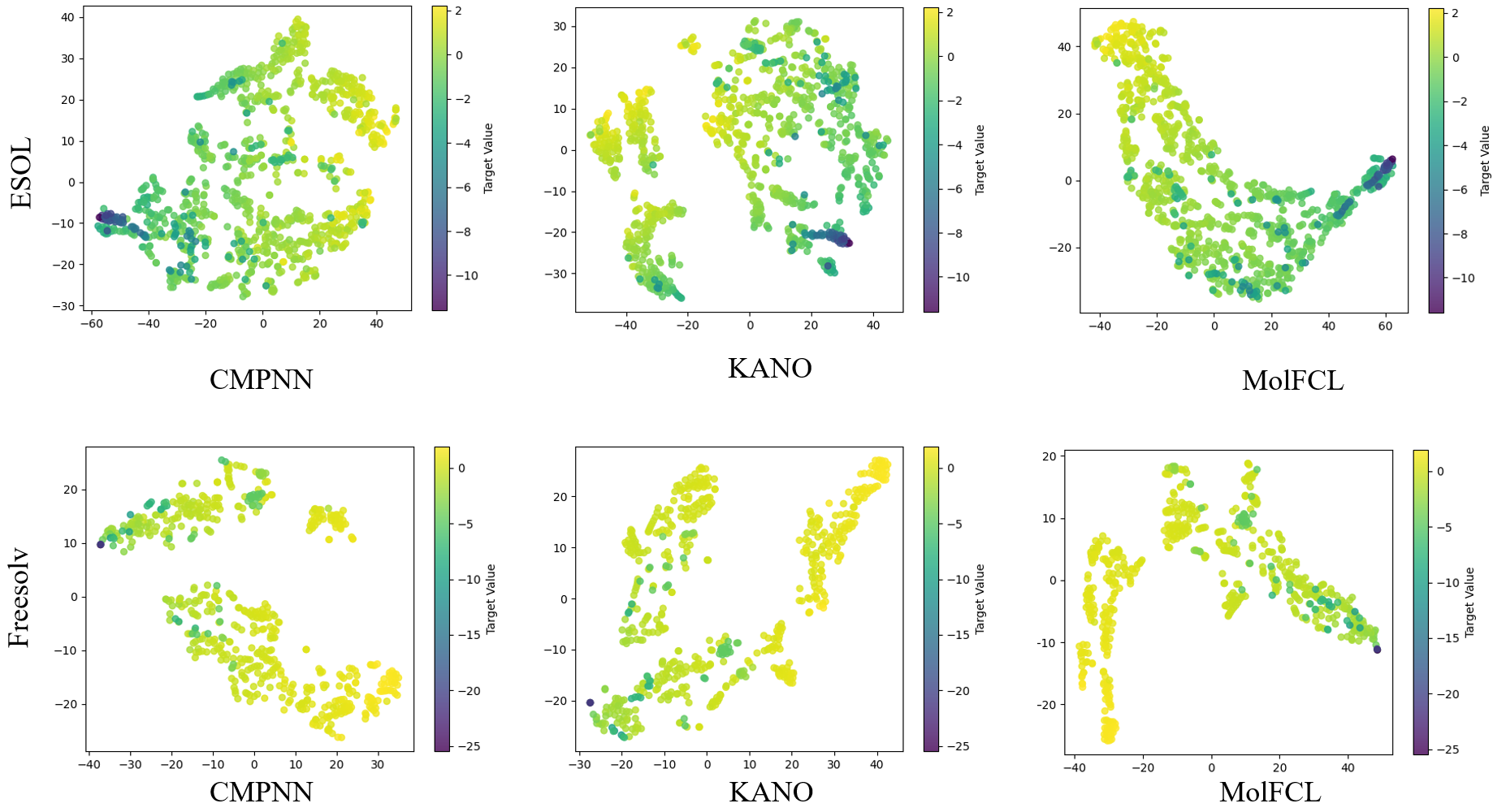


**Supplementary Figure S4.** We present t-SNE visualizations of embeddings for examples with continuous property values in regression tasks. Compared to the best baseline, our model can learn smoother embedding representations within molecules with continuous property values.


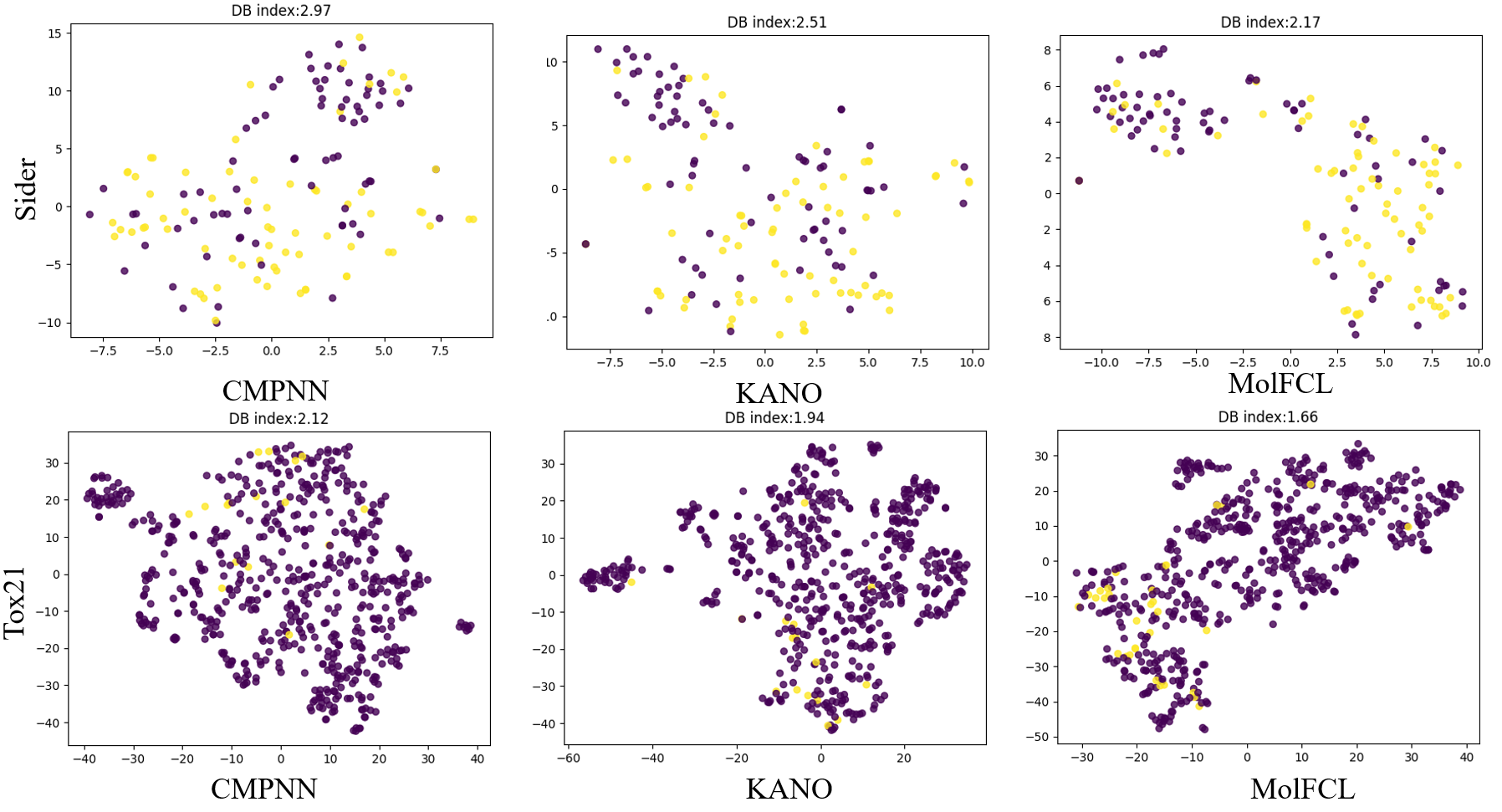


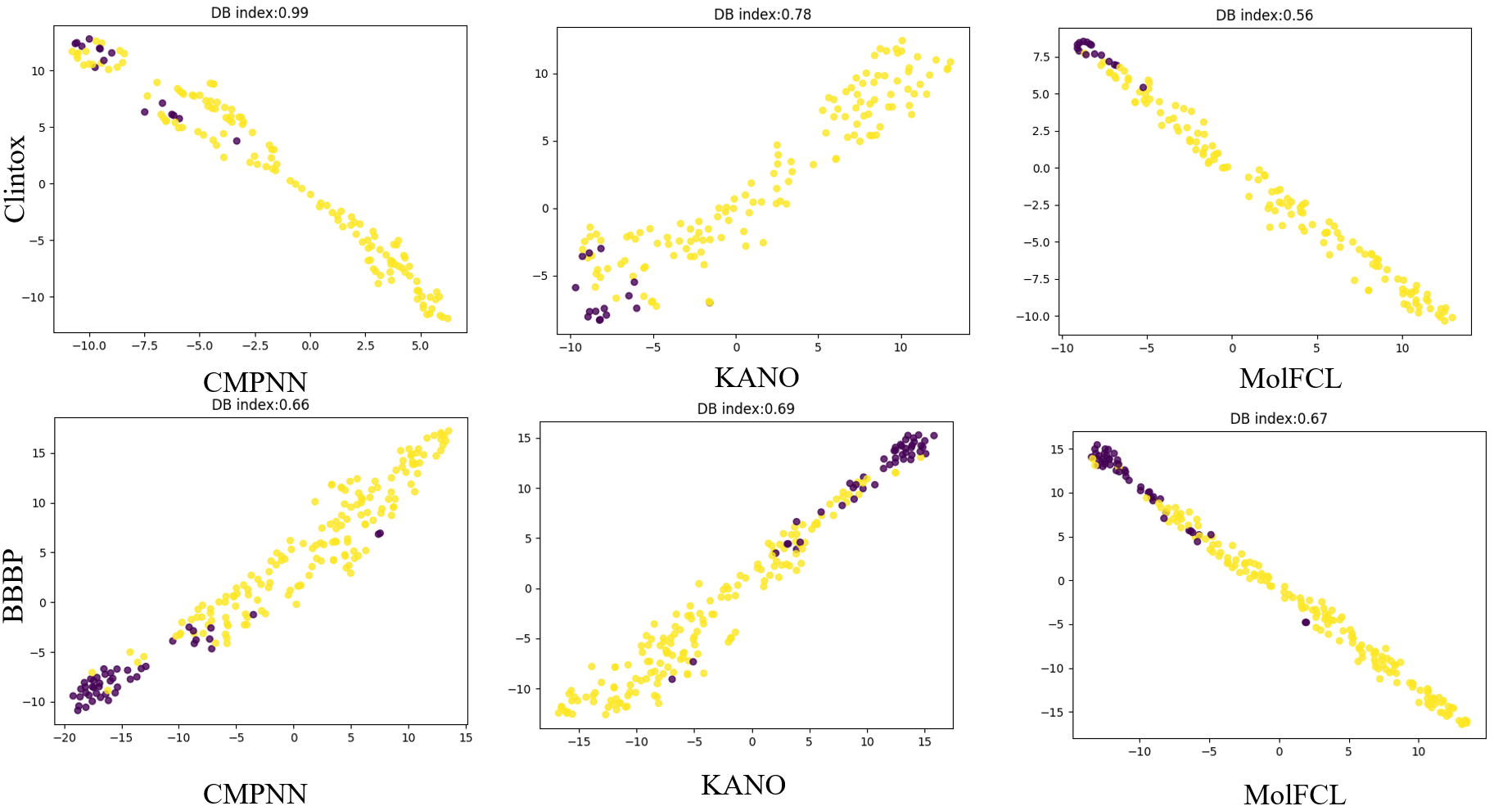


**Supplementary Figure S5.** To assess the ability of MolFCL to learn excellent representations in unknown molecules, we use the test set to examine the molecular representations extracted by different models. As shown in the figure above, excep t for the BBBP dataset, MolFCL can generate more distinctive clusters and has the lowest DB index.

1. **Cluster analysis in BACE prediction**

in Supplementary Figure S7A, our model divides the yellow points (positive samples) into two clusters, where we define the lower cluster as Cluster 1 and the upper cluster as Cluster 2. We apply the BRICS algorithm to fragment Cluster 1 and Cluster 2, respectively. By counting the fragment occupancy, we find that aromatic rings have a higher occupancy in Cluster 1 as shown in Figure R2B. The work of Galeana-Ascencio R A et al. states [1] that in BACE prediction, the aromatic ring is more likely to interact with the hydrophobic pocket around the Ile109 residue in BACE-1, thus acting as an inhibitor. As a result, Cluster 1 has a more pronounced difference in the distribution of features compared to Cluster 2 with respect to the negative samples.


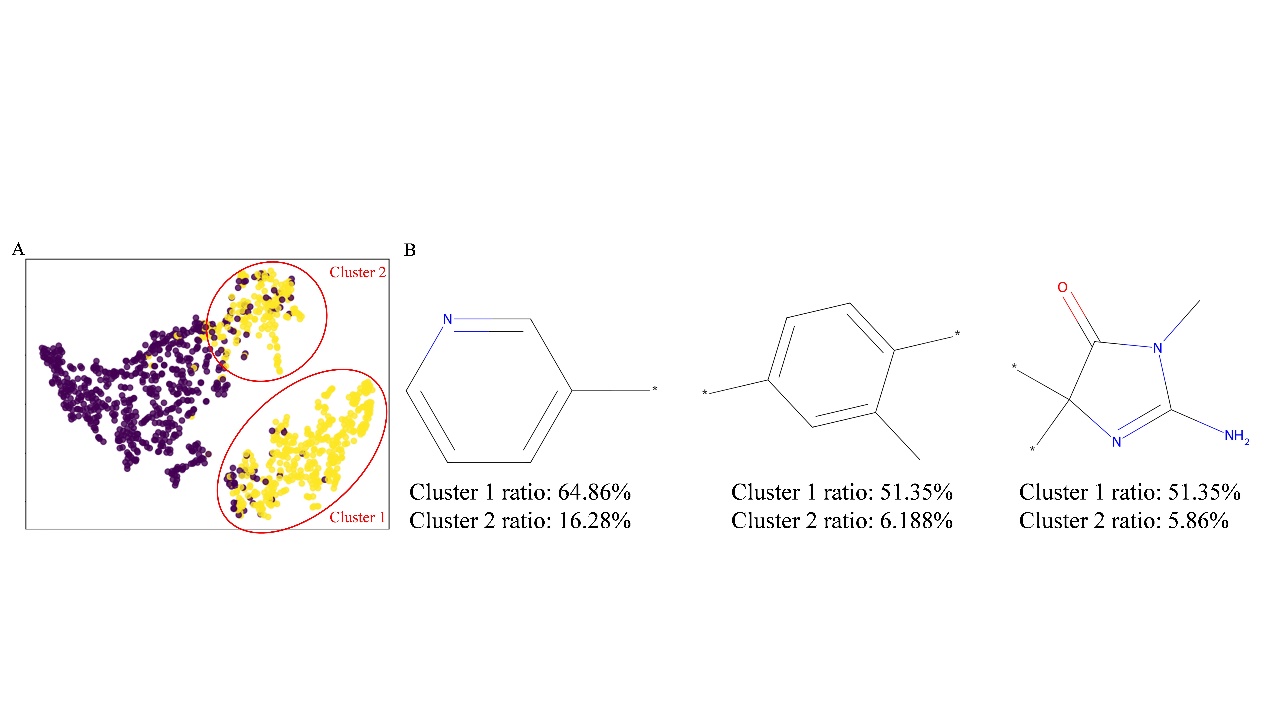


**Supplementary Figure S6**. Visual difference analysis of two clusters. (A) Molecular regions contained in clusters 1 and 2. (B) Percentage of aromatic rings in Cluster 1 and Cluster 2.

[1] Galeana-Ascencio R A, Mendieta L, Limon D I, et al. β-Secretase-1: In Silico Drug Reposition for Alzheimer’s Disease[J]. International Journal of Molecular Sciences, 2023, 24(9): 8164.

1. **Case study**


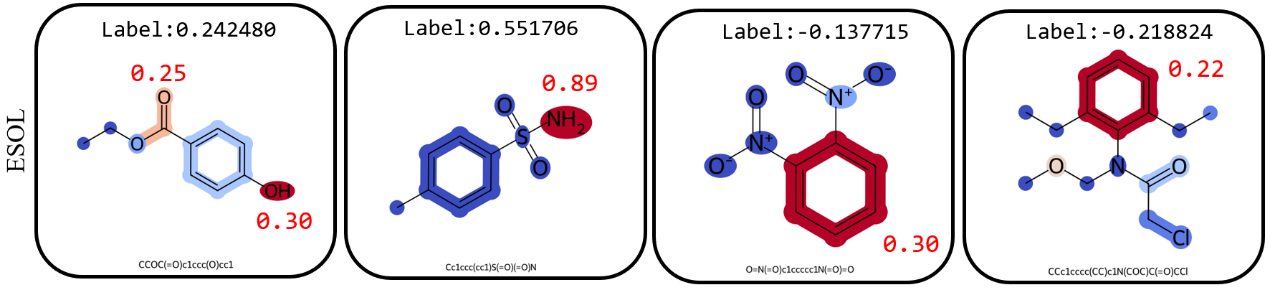


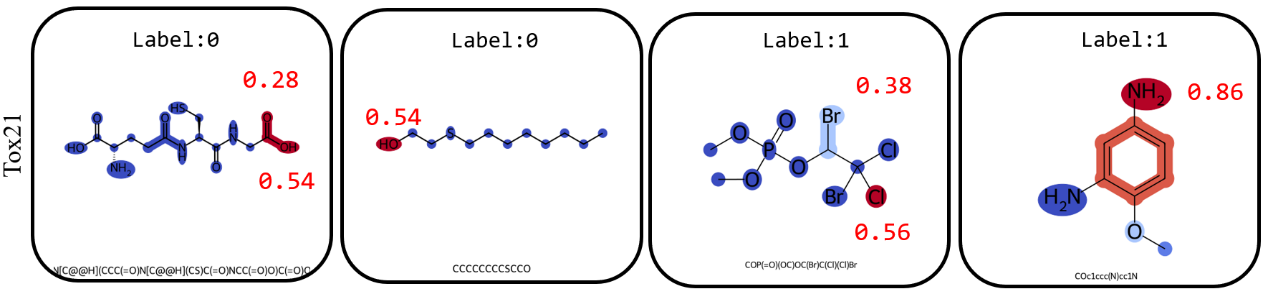


**Supplementary Figure S7. Interpretability of prompt learning.** Examining the weight allocation of MolFCL for different functional group substructures in eight molecules from two datasets. Higher weights indicate greater importance of the structure, with darker colors representing higher weights.

1. **Ablation Study**

**Supplementary Table** **S7.** Results of ablation experiments on nine benchmark datasets. We perform three independent runs on the pre-trained model, reporting their average and standard deviation. ROC-AUC is used as the evaluation metric for classification tasks, while RMSE serve as the metric for regression tasks.

| Models | Classification (ROC-AUC, higher is better↑) | | | | | | | Regression (RMSE, lower is better↓) | | | |
| --- | --- | --- | --- | --- | --- | --- | --- | --- | --- | --- | --- |
|  | BBBP | Tox21 | Toxcast | Sider | Clintox | Bace | AVG | ESOL | Freesolv | Lipo | AVG |
| Original CMPNN | 0.927  (0.017) | 0.801  (0.016) | 0.708  (0.013) | 0.616  (0.003) | 0.898  (0.008) | 0.867  (0.002) | 0.803 | 0.798  (0.112) | 1.570  (0.442) | 0.614  (0.029) | 0.994 |
| w/o MolFCL pretraining | 0.969  (0.004) | 0.830  (0.010) | 0.723  (0.014) | 0.619  (0.18) | 0.930  (0.013) | 0.917  (0.012) | 0.831 | 0.708  (0.025) | 1.322  (0.135) | 0.621  (0.027) | 0.884 |
| w/o MolFCL atom message | 0.974  (0.002) | 0.837  (0.011) | 0.735  (0.013) | 0.638  (0.019) | 0.945  (0.011) | 0.912  (0.011) | 0.840 | 0.663  (0.023) | 1.156  (0.128) | 0.611  (0.021) | 0.810 |
| w/o MolFCL FP | 0.978  (0.001) | 0.840  (0.008) | 0.736  (0.015) | 0.660  (0.021) | 0.953  (0.014) | **0.938**  **(0.009)** | 0.950 | 0.656  (0.022) | 1.065  (0.102) | 0.599  (0.004) | 0.773 |
| MolFCL | **0.981**  **(0.002)** | **0.841**  **(0.015)** | **0.741**  **(0.015)** | **0.660**  **(0.017)** | **0.955**  **(0.015)** | 0.933  (0.010) | **0.952** | **0.638**  **(0.024)** | **1.045**  **(0.160)** | **0.601**  **(0.034)** | **0.761** |

**Supplementary Table S8.** Runtime of each variant on different datasets.

| Models | Runtime (mins-secs) | | | | | | | | |
| --- | --- | --- | --- | --- | --- | --- | --- | --- | --- |
|  | BBBP | Tox21 | Toxcast | Sider | Clintox | Bace | ESOL | Freesolv | Lipo |
| Variant1 | 27 - 17 | 102 - 8 | 113 - 24 | 27 - 15 | 21 - 52 | 26 - 35 | 7 - 21 | 3 - 1 | 58 - 25 |
| Variant2 | 119 - 9 | 340 - 57 | 369 - 18 | 106 - 32 | 80 - 6 | 117 - 4 | 33 - 21 | 20 - 9 | 296 - 32 |
| Variant3 | 128 - 1 | 349 - 14 | 405 - 6 | 132 - 45 | 73 - 4 | 115 - 11 | 32 - 20 | 12 - 6 | 315 - 33 |
| Variant4 | 127 - 54 | 347 - 34 | 401 - 2 | 133 - 7 | 73 - 6 | 112 - 41 | 33 - 50 | 12 - 11 | 313 - 13 |
| MolFCL | 142 - 48 | 421 - 32 | 477 - 22 | 135 - 32 | 79 - 5 | 133 - 21 | 40 - 13 | 17 - 6 | 322 - 56 |

*Note that variant1 is the Original CMPNN, variant2 is the w/o MolFCL pretraining, variant3 is the w/o MolFCL atom message, and variant4 is the w/o MolFCL FP.

1. **Chemical Descriptors Prompt**

To evaluate whether fine-tuning based on functional groups outperforms the combination of chemical descriptors such as ECFP, we conducted corresponding experiments. Specifically, we have replaced functional group-guided learning with molecular ECFP fingerprints and conducted the experiments accordingly, where the fingerprint's minPath was set to 1, maxPath to 7, and the fingerprint vector length was set to 300. The results, presented in Supplementary Table S9, demonstrate that fine-tuning based on functional groups consistently achieves better performance compared to ECFP descriptors. This improvement arises from the ability of functional groups to provide finer-grained information combinations. Furthermore, our carefully designed model architecture enables the selective emphasis on key functional group information while attenuating the influence of less critical groups. In contrast, ECFP descriptors encode information from the entire molecule at a coarser granularity, making it difficult to bridge the gap between pretraining tasks and downstream fine-tuning tasks.

**Supplementary Table S9.** The performance of MolFCL when replacing the functional group prompt with the ECFP prompt.

| Models | Classification (ROC-AUC, higher is better↑) | | | | | | Regression (RMSE, lower is better↓) | | |
| --- | --- | --- | --- | --- | --- | --- | --- | --- | --- |
|  | BBBP | Tox21 | Toxcast | Sider | Clintox | Bace | ESOL | Freesolv | Lipo |
| MolFCL  (ECFP prompt) | 0.973  (0.002) | 0.835  (0.015) | 0.735  (0.011) | 0.629  (0.021) | 0.942  (0.014) | **0.937**  **(0.012)** | 0.780  (0.091) | 1.866  (0.154) | 0.633  (0.015) |
| MolFCL(functional group prompt) | **0.981**  **(0.002)** | **0.841**  **(0.015)** | **0.741**  **(0.015)** | **0.660**  **(0.017)** | **0.955**  **(0.015)** | 0.933  (0.010) | **0.638**  **(0.024)** | **1.045**  **(0.160)** | **0.601**  **(0.034)** |
